# The Relationship between Epicardial Adipose Tissue Thickness and Serum Interleukin-17a Level in Patients with Isolated Metabolic Syndrome

**DOI:** 10.3390/biom9030097

**Published:** 2019-03-11

**Authors:** Esra Demir, Nazmiye Özlem Harmankaya, İrem Kıraç Utku, Gönül Açıksarı, Turgut Uygun, Hanise Özkan, Bülent Demir

**Affiliations:** 1 Department of İnternal Medicine, Kanuni Sultan Süleyman Education and Research Hospital, 34103 İstanbul, Turkey; esracokicli@hotmail.com (E.D.); iremkrac@yahoo.com (İ.K.U.); haniseozkan@hotmail.com (H.Ö.); 2 Department of İnternal Medicine, Bakırköy Dr Sadi Konuk Education and Research Hospital, 34147 İstanbul, Turkey; oharmankaya@yahoo.com; 3 Department of Cardiology, Medeniyet University Medical Faculty, 34722 İstanbul, Turkey; drgonulkutlu@hotmail.com; 4 Department of Cardiology, Konya Education and Research Hospital, 42040 Konya, Turkey; turgutuyguncapa@hotmail.com; 5 Department of Cardiology, Bakırköy Dr Sadi Konuk Education and Research Hospital, 34147 İstanbul, Turkey

**Keywords:** metabolic syndrome, adipose tissue, interleukin 17A

## Abstract

In this study, it was aimed to investigate the relationship between the epicardial adipose tissue thickness (EATT) and serum IL-17A level insulin resistance in metabolic syndrome patients. This study enrolled a total of 160 subjects, of whom 80 were consecutive patients who applied to our outpatient clinic and were diagnosed with metabolic syndrome, and the other 80 were consecutive patients who were part of the control group with similar age and demographics in whom the metabolic syndrome was excluded. The metabolic syndrome diagnosis was made according to International Diabetes Federation (IDF)-2005 criteria. EATT was measured with transthoracic echocardiography (TTE) in the subjects. IL-17A serum levels were determined using the ELISA method. Fasting blood glucose, HDL, triglyceride, and fasting insulin levels were significantly higher in the metabolic syndrome group compared to the control group. In addition, the metabolic syndrome group had significantly higher high-sensitivity C-reactive protein (hs-CRP) and Homeostatic Model Assessment Insulin Resistance (HOMA-IR) levels than the control group. Similarly, serum IL-17A levels were significantly elevated in the metabolic syndrome group compared to the control group statistically (*p* < 0.001). As well, EATT was higher in the metabolic syndrome than the control group. Conclusion: By virtue of their proinflammatory properties, EATT and IL-17 may play an important role in the pathogenesis of the metabolic syndrome.

## 1. Introduction

Metabolic syndrome is defined as a constellation of risk factors, mainly visceral obesity accompanied by hypertension, dyslipidemia, and impaired fasting glucose. It’s prevalence has been gradually increasing in parallel to an increase in the prevalence of obesity [1]. The importance of metabolic syndrome stems from an excess risk of type 2 diabetes mellitus (DM) and coronary artery disease [2]. Therefore, it has transformed into an important public health problem. Hence, it is of paramount importance to understand its pathogenesis and treat affected patients before it progresses to chronic disorders. Visceral obesity and associated insulin resistance play an important role for the development of metabolic syndrome. Furthermore, visceral adipose has been shown to behave differently than subcutaneous adipose and to release many cytokines and adipokines that cause the metabolic syndrome. [3]. Additionally, low-grade inflammation plays an important role for the pathogenesis of the metabolic syndrome and coronary artery disease. Hence, many studies to date have shown higher high-sensitive C-reactive protein (hs-CRP) levels, reflecting inflammation among patients with metabolic syndrome [4]. In patients with metabolic syndrome, inflammation induces insulin resistance and atherosclerosis through a variety of mechanisms [3].

Epicardial adipose tissue (EAT) is a space between the visceral pericardium and the outer margin of myocardium, whose thickness can be measured by transthoracic echocardiography (TTE). Epicardial adipose tissue (EAT) is considered an important component of chest adipose tissue, and an increasing number of studies have been conducted about it. EAT appears to play a particularly important role for the pathogenesis of coronary artery disease [5]. By virtue of its proximity to coronary arteries, EAT exerts local and parenchymal effects that effectively contribute to the development of atherosclerosis. That is, by its proximity to coronary arteries, EAT contributes to the stimulation of local inflammation that plays an important role for the initiation, progression, and complications of atherosclerosis. It has also been shown that EATT is increased in obese patients and patients with metabolic syndrome compared to the normal population [6,7]. Hence, EAT, an important component of visceral adipose tissue, appears to exacerbate inflammation and to contribute to the development of coronary artery disease and insulin resistance among patients with metabolic syndrome.

Interleukin-17 (IL-17) is a relatively novel cytokine released by helper T cells-17 (T-helper 17), which has marked proinflammatory properties. Studies have shown that the IL-17 family has several subtypes, the most important one of which appears to be IL-17A. There is scientific evidence that IL-17 plays an important role for the development of coronary artery disease and acute coronary syndromes [8,9]. Similarly, some studies have shown that IL-17 may play a role for the pathogenesis of various autoimmune disorders and DM [10,11]. A review of the literature about metabolic syndrome revealed that there is a small body of knowledge of the relationship between metabolic syndrome and IL-17. However, by virtue of its proinflammatory properties, IL-17 may play an important role for the pathogenesis of the metabolic syndrome whose pathogenesis involves inflammation.

In the light of these data, our study aimed to investigate the relationship between EAT and serum IL-17 and insulin resistance among patients with isolated metabolic syndrome, that is without coronary artery disease or DM. To our knowledge, this subject has not been yet studied in the literature.

## 2. Materials and Methods

### 2.1. Study Design and Patient Selection

Our study was designed as a prospective, observational case-control study. Our study included 80 consecutive patients (23 male, 57 females; mean age 39.0 ± 11.5 years) who presented to the endocrinology, diabetes, and obesity or internal medicine outpatient clinics and who were diagnosed with metabolic syndrome. The control group consisted of 80 patients (16 males, 64 females; mean age 39.6 ± 9.4 years) having similar ages and demographic characteristics but no metabolic syndrome. 

The exclusion criteria included having a previous or newly diagnosed diabetes mellitus, documented coronary artery disease, acute coronary syndrome, peripheral artery disease or carotid artery stenosis, previous cerebrovascular events, myocarditis, pericarditis, cardiomyopathy, congestive heart failure, congenital heart disease, moderate-to-severe cardiac valvular disease, atrial fibrillation, active infection, autoimmune disease, active or chronic hepatitis or liver disease, impaired renal function (serum creatinine > 1.5 mg/dL or above), chronic inflammatory disease, anti-inflammatory or immune suppressive medication use. Detailed anamnesis was taken from each patient. Cardiovascular risk factors, namely, smoking status, hyperlipidemia, hypertension, diabetes mellitus, and family history for cardiovascular disease were questioned.

All subjects underwent detailed physical examination. Additionally, all patients were examined with twelve-lead electrocardiography. All subjects’ weight and height were measured and recorded. Body mass index was calculated using the formula weight (kg) /height(m)^2^. Waist circumference was measured from the narrowest horizontal distance between the lowermost rib and the anterior iliac spinal process while the patients were in upright position with their waist open.

Diabetes mellitus was defined as a fasting blood glucose level of 126 mg/dL or higher, or being on antidiabetic treatment. Patients with a plasma glucose of 100 mg/dL to 126 mg/dL were said to have impaired fasting glucose. Hypertension was defined as resting systolic blood pressure of 140 mmHg or higher or resting diastolic blood pressure of 90 mmHg or higher in at least three measurements, or being on antihypertensive medication.

Hyperlipidemia was defined as a total cholesterol level of 200 mg/dL or higher, or a triglyceride level of 150 mg/dL or higher [12].

Patients who were actively smoking were considered smokers irrespective of the number of cigarettes ever smoked.

Family history for coronary artery disease was defined as having a male relative with coronary artery disease younger than 55 years of age or having a female relative with coronary artery disease younger than 65 years of age.

Metabolic syndrome was diagnosed on the basis of the International Diabetes Federation 2005 Criteria [13]. It was defined as abdominal obesity, defined as a waist circumference of > 94 cm in men and > 80 cm in women, plus at least two of the criteria below:

1. High Triglyceride level: ≥ 150 mg/dL (1.7 mmol/L).
2. Low HDL cholesterol: < 40 mg/dL (1.03 mmol/L) in men and < 50mg/dL (1.29 mmol/L) in women.
3. High blood pressure: Systolic blood pressure ≥130 mmHg or diastolic blood pressure ≥ 85 mmHg.
4. Increased fasting plasma glucose: Fasting plasma glucose ≥ 100 mg/dL (5.6 mmol/L).

### 2.2. Measurement of Epicardial Adipose Tissue Thickness by Echocardiography

All study subjects underwent detailed two-dimensional, M-mode and Doppler echocardiographic examination by two experienced echocardiographers who were unaware of the biochemical parameters. Echocardiographic examinations were performed with a Vivid S-5 (GE Vingmed, Horten, Norway) device using a 2.5 MHz–3.5 MHz probe. Epicardial adipose tissue was defined as the adipose tissue between the pericardium and the outer margin of the myocardium [14]. In echocardiography EAT is seen as a relatively echo-free space between the outer margin of the myocardium and the visceral layer of the pericardium. In compliance with the previous reports, we measured epicardial adipose tissue thickness perpendicular to the right ventricular free wall, from the parasternal long axis and transverse views at end-diastole [14,15]. Aortic annulus and interventricular septum were used as anatomic markers to ensure perpendicularity to the right ventricular free wall [14]. In order to reduce the margin of error, final epicardial adipose thickness was calculated from the mean value of three measurements in consecutive cardiac cycles.

### 2.3. Laboratory Parameters

For biochemical analyses, blood samples were drawn from the forearm veins into dry tubes containing gel after 12 h of fasting. The samples were then waited for 30 min and centrifuged at 4000 rpm for 10 min. The serum sample obtained from the first tube was then transferred into an eppendorf and stored at −80 degrees for IL-17 measurement. Serum samples obtained from other tubes were studied on the same day for routine biochemistry analyses with Beckman Coulter AU5800 biochemistry autoanalyser and for hormone tests with Beckman Coulter UniCel DxI800 Immunoassay autoanalyser with original kits (Beckman Coulter Inc, West Sacramento, California, USA). Insulin level was measured with Siemens Immulite 2000 analyser and original kits, using and the chemiluminescence method (Siemens Healthcare, Erlangen, Germany). Complete blood count analysis was performed from the blood samples taken into ethylenediaminetetraacetic acid (EDTA) containing tubes, using a Beckman Coulter LH 750 analyser.

### 2.4. Determination of Serum IL-17A Level

After completing collection of all blood samples, stored at −80 °C. Serum samples were thawed to room temperature for Il-17 analysis. IL-17 level was quantitatively determined with Diaclone Human IL-17A ELISA Kit (catalog 850.940.096, Lot 117A-12), using ELISA (Enzyme –Linked Immunosorbent Assay) method based on the sandwich method, in compliance with the procedure recommended by the manufacturer. The results of were reported as pg/ml. The intraassay and intraassay coefficients of variation, reflecting the measurement’s specific performance properties, were determined as 3.3% and 5.2%, respectively.

### 2.5. Other Parameters

Serum hs-CRP level was measured nephelometrically, using a BN-II nephelometer (Siemens Healthcare Diagnostics, Los Angeles, CA, USA). Insulin resistance was calculated using the homeostasis model assessment insulin resistance index (HOMA-IR) formula [16].

This study was conducted in compliance with the Helsinki Declaration and approved by the ethics committee of our hospital (Decision Number: 2013/09/01). All subjects gave written informed consent before study entry.

### 2.6. Statistical Analysis

Study data were analysed using Windows (SPSS Inc, Chicago, IL, USA) SPSS software package, version 22.0. Descriptive statistics included frequency, ratio, mean, median, minimum, maximum, and standard deviation. Normality of distribution was tested with Kolmogorov-Smirnov test. Quantitative data were analyzed using independent samples T test and Mann-Whitney U test. Qualitative data were analyzed using Chi-Square test. Correlation analyses were performed with Spearman correlation analysis. Multivariable linear regression analysis was performed to determine the level of effect. Standard beta coefficients and 95% confidence intervals were calculated. Receiver operator characteristics (ROC) curve analysis was done to calculate cut off levels for differentiating patients with metabolic syndrome. Statistical significance was set at *p* < 0.05.

## 3. Results

Patient’s demographic data, clinical properties, and laboratory parameters were summarized on Table 1. There was no significant difference between the metabolic syndrome and control groups with respect to age, sex, and rate of smoking (*p* > 0.05). The metabolic syndrome group had significantly higher rates of hypertension, hyperlipidemia, and family history for coronary artery disease, as well as numerical value BMI, systolic blood pressure, and diastolic blood pressure (*p* < 0.001).

The comparison of the laboratory’s parameters and echocardiographic parameters revealed that fasting plasma glucose, HDL, Triglyceride, GGT, AST, ALT and fasting insulin levels were significantly greater in the metabolic syndrome group (for all comparisons, *p* < 0.01). As for insulin resistance, the comparison of HOMA-IR values between the two groups showed that the metabolic syndrome group had a significantly greater HOMA-IR level compared to the control group (5.2 ± 2.3 vs. 1.4 ± 0.6, *p* < 0.001) (Table 1).

Similarly, serum hs-CRP level was significantly greater in the metabolic syndrome group compared to the control group (3.8 ± 0.6 mg/L vs. 0.8 ± 0.8 mg/L, *p* < 0.001) (Table 1).

**Figure 1:**
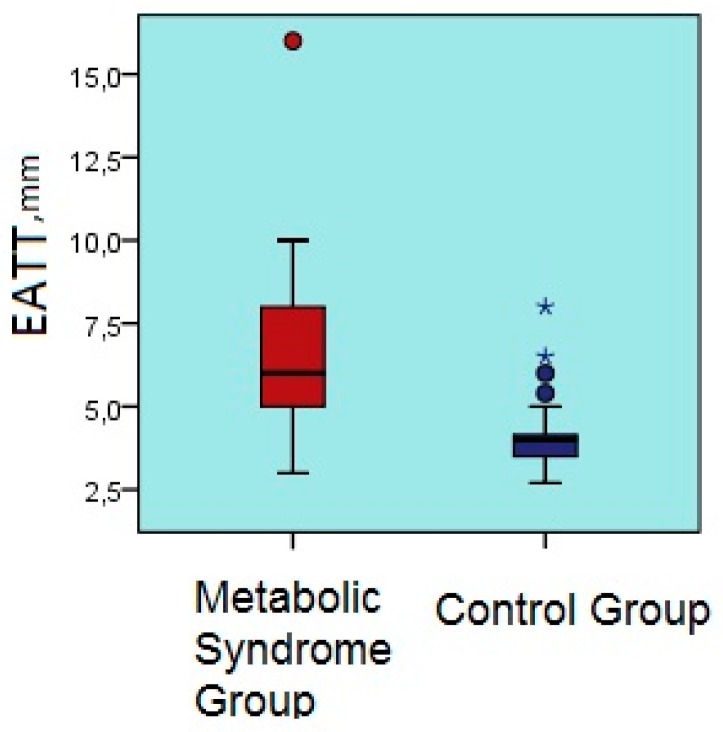
Comparison of epicardial adipose tissue thicknesses of the metabolic syndrome and control groups. EATT, epicardial adipose tissue thickness.

Epicardial adipose tissue thickness was significantly greater in patients with metabolic syndrome than the controls (6.2 ± 2.2 mm vs. 4.0 ± 0.8 mm, *p* < 0.001) (Table 1) (Figure 1).

**Figure 2:**
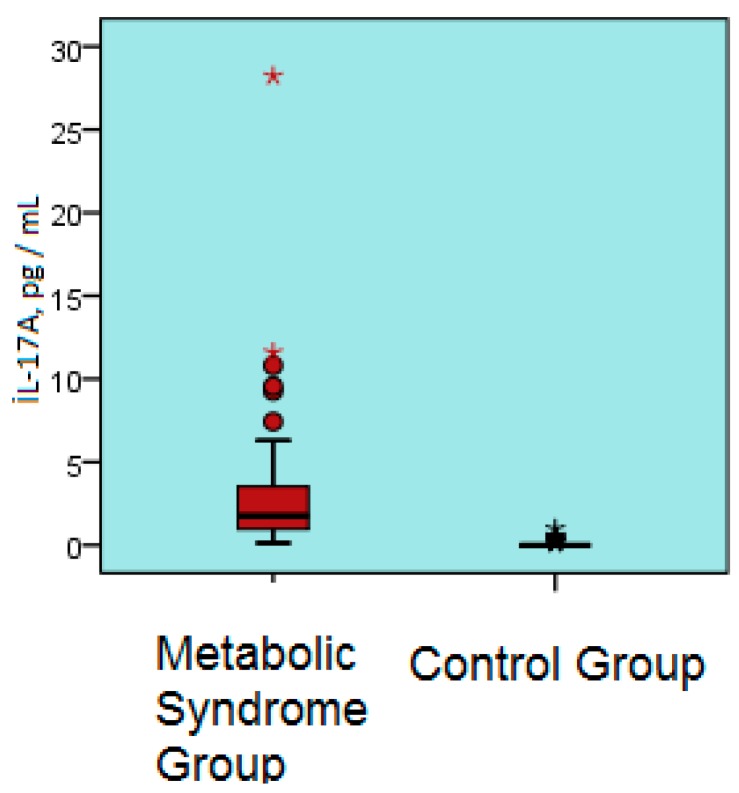
Serum IL-17A levels of the metabolic syndrome and control groups. IL-17A; interleukin 17A, pg/ mL.

Serum interleukin 17A level was significantly greater in the metabolic syndrome group compared to the control group (2.9 ± 3.7 pg/mL vs. 0.1 ± 0.2 pg/mL, *p* < 0.001) (Table 1) (Figure 2).

**Figure 3:**
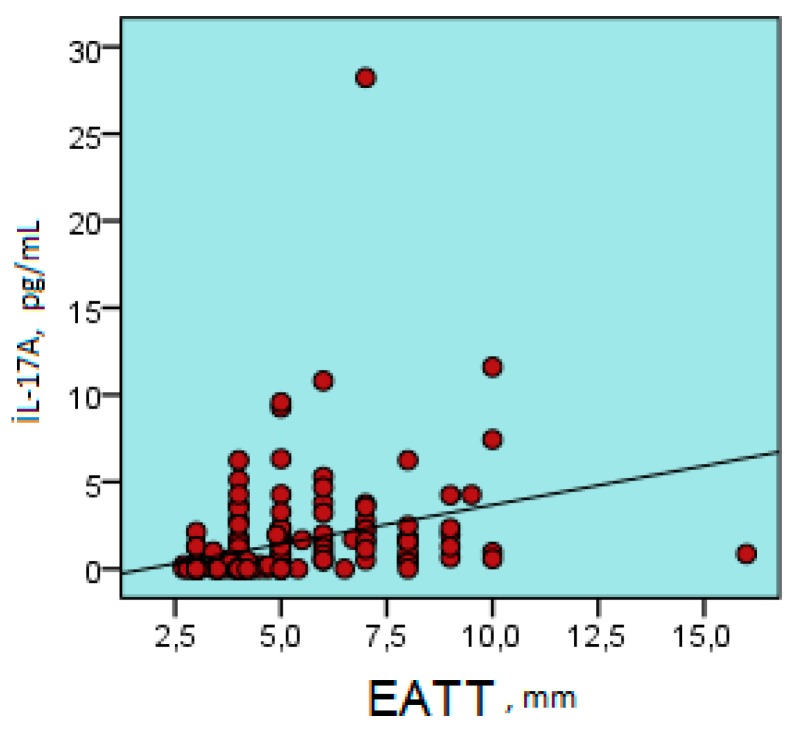
Positive correlation between epicardial adipose tissue thickness and serum IL-17A level (r = 0.308, *p* < 0.001). IL-17A; interleukin 17A, pg/ mL; EATT, Epicardial Adipose Tissue Thickness.

**Figure 4:**
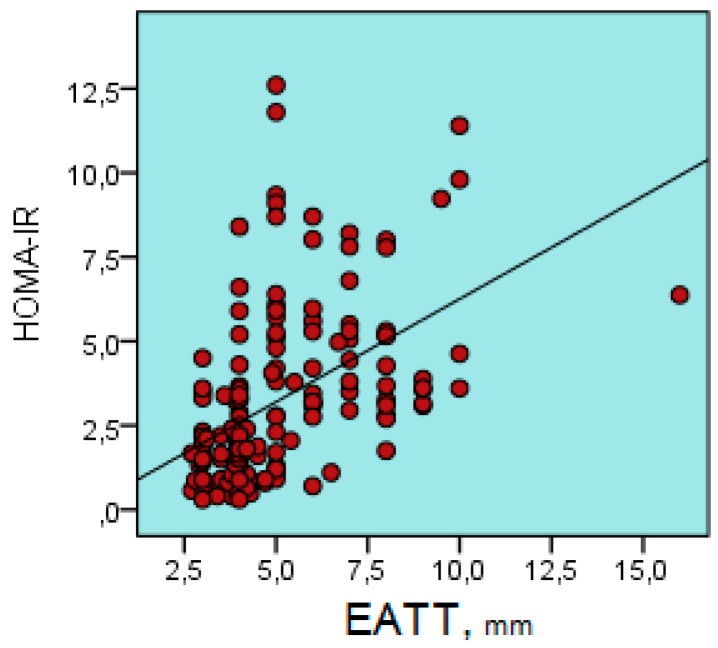
Positive correlation between epicardial adipose tissue thickness and HOMA-IR (r = 0.567, *p* < 0.001). HOMA-IR, Homeostasis Model Assessment İnsulin Resistance Index; EATT, Epicardial Adipose Tissue Thickness.

There was a positive correlation between EATT and age, BMI, glucose level, insulin, Tg, GGT, HOMA-IR, hs-CRP, IL-17A, urea, systolic blood pressure, and diastolic blood pressure ( r = 0.180, r = 0.539, r = 0.194, r = 0.279, r = 0.556, r = 0.281, r = 0.666, r = 0.308, r = 0.181, r = 0.421, and r = 0.394, respectively; for all comparisons, *p* < 0.05) (Table 2) (Figure 3 and Figure 4).

A multivariable linear regression analysis showed that EATT was significantly correlated to age, diastolic blood pressure, HOMA-IR and urea (for all comparisons, *p* < 0.05) (Table 3).

### The Predictive Values of Epicardial Adipose Tissue Thickness and Interleukin 17A Level for Metabolic Syndrome

**Figure 5:**
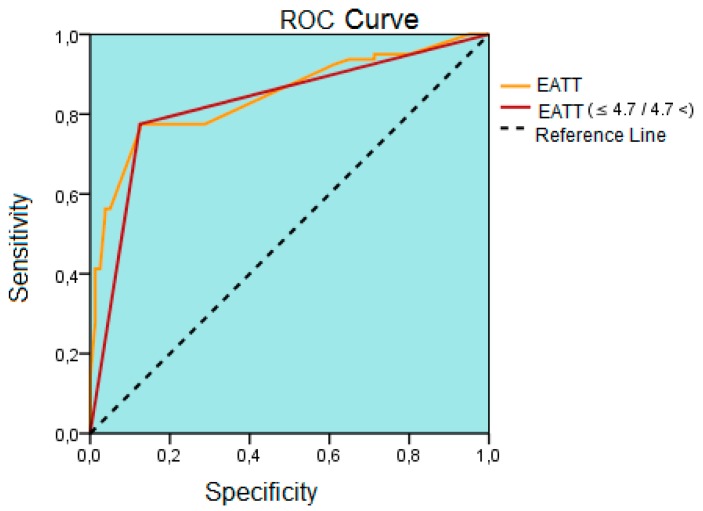
The result of the receiver operating characteristics (ROC) curve analysis drawn to determine the best cutoff point of epicardial adipose tissue thickness to differentiate patients with metabolic syndrome. ROC; receiver operating characteristics, EATT; epicardial adipose tissue thickness, mm; millimeter.

The receiver operator characteristics (ROC) curve analysis showed that values above a cutoff level of 4.7 mm for EATT had a sensitivity of 77.5%, a specificity of 87.5%, a positive predictive value of 86.1%, and a negative predictive value of 79.5% for metabolic syndrome (area under the curve; 0.83, 95% Confidence Interval: 0.76–0.89, *p* < 0.001) (Figure 5).

## 4. Discussion

The main finding of our study was that the EATT and IL-17A level were higher among patients with metabolic syndrome compared to the control group. The other important finding of our study was that there was a positive correlation between serum IL-17 A level and EATT and insulin resistance. According to our knowledge, our study is the first to scrutinize the relationship between IL-17A, EAT, and insulin resistance.

Metabolic syndrome is a cluster of risk factors, the prevalence of which has been gradually increasing. Visceral obesity is of particular importance to the development of the metabolic syndrome. As is well known, EAT is an important component of adipose tissue. We detected a greater EATT among patients with metabolic syndrome than the control group, which is evidence of increased chest adipose accumulation. This finding was further supported by a positive correlation between BMI and EATT. In parallel to our findings, a sufficient number of studies have shown that EATT increased among patients with metabolic syndrome [17,18,19,20]. Increased EAT may contribute to increased incidence of coronary artery disease in this population because visceral adipose tissue is a metabolically active and may contribute to atherosclerosis by synthesizing many adipocytokines. Furthermore, the close proximity of EAT to coronary arteries suggests that it may contribute to the appearance of atherosclerosis not only by its systemic actions, but also by local and paracrine effects. Low-grade inflammation is particularly effective for the development of atherosclerosis. As EAT is a component of chest adipose tissue, it is expected to play a role for the inflammatory changes. Hence, Mazurek et al. showed that human epicardial adipose tissue is an important source of inflammatory mediators [21]. In that study, they showed that IL-1, IL-6, IL-6sR, and TNF-alpha expression was greater in epicardial adipose tissue than that in subcutaneous adipose tissue among patients with coronary artery disease [21]. Interestingly, that same study failed to detect any increase in the serum levels of the cytokines with increased EAT expression [21]. In the light of all the above mentioned data, it becomes clear that EAT has local proinflammatory effects. In another study by Iacobellis et al. where biopsy samples taken from epicardial adipose tissue among patients undergoing elective cardiac surgery, a significantly reduced adiponectin level was detected by the Western Blot method among patients with coronary artery disease [22]. Adiponectin is an important adipokine known to have anti-inflammatory and antiatherosclerotic properties. Detection of a lower adiponectin level in epicardial adipose tissue in patients with coronary artery disease suggests that epicardial adipose tissue impairs anti-inflammatory/proinflammatory balance in favor of proinflammation, contributing to atherosclerosis by inducing local inflammation. Similarly, Hirata et al., in a study on EAT biopsy samples obtained from patients undergoing elective cardiac surgery, demonstrated that macrophage polarization shifted towards proinflammation in epicardial adipose tissue in patients with coronary artery disease [23]. They reach that conclusion by demonstrating a higher M1/M2 ratio, reflecting proinflammatory and anti-inflammatory state in macrophages, among patients with coronary artery disease than those without [23]. Furthermore, they also found a positive correlation between the expression of proinflammatory cytokines and M1/M2 ratio in EAT but found a negative correlation between the expression of anti-inflammatory cytokines and the M1/M2 ratio [23]. This supports the notion that EAT induces marked inflammation and thus plays an active role for the development of atherosclerosis. Another interesting finding of the present study was the absence of any significant difference between patients with and without coronary artery disease with respect to macrophage infiltration and cytokine expression in subcutaneous adipose tissue [23]. This supports the view that EAT is an important component of chest adipose tissue and paves the way for inflammation, which actively plays a role in the pathogenesis of many cardiometabolic disorders. 

As is known, one of the major diagnostic criteria of metabolic syndrome is increased waist circumference reflecting visceral obesity. Increased EATT among patients with metabolic syndrome is a reflection of visceral obesity. Hence, clinical studies among obese persons found a significant positive correlation between EAT and obesity [24,25]. Willens et al. showed a significant reduction in EATT in parallel to total weight loss among patients undergoing bariatric surgery [26]. All those data suggest that EAT is an important component of visceral obesity. We found a positive correlation between EATT and BMI, further supporting this hypothesis. 

One of the main problems with metabolic syndrome is insulin resistance and its clinical consequences, that is impaired fasting glucose and type 2 DM. Therefore, as a component of visceral obesity, EAT may play a role for the development of insulin resistance. Iacobellis et al., in a study on non-diabetics, found a significant positive correlation between EATT and impaired fasting glucose [27]. The same research also showed a significant correlation between EATT and fasting glucose [28]. A recent study on postmenopausal women showed a correlation between EAT and visceral adipose tissue, metabolic syndrome, and insulin resistance [29]. 

Another important finding of our study is that the IL-17A level was significantly greater among patients with metabolic syndrome compared to the control subjects. Interleukin 17A is a relatively novel cytokine that is released by lymphocytes called T-17 and known to have proinflammatory effects. There is limited information in the literature as to the relationship between metabolic syndrome and IL-17. The most important of these data comes from the study by Surendar et al. on patients with metabolic syndrome [30]. That study reported a lower serum IL-17A level among patients with metabolic syndrome compared to the controls [30]. That same study found that the serum transforming growth factor (TGF) β level was higher among patients with metabolic syndrome than the control subjects [30]. This is an unexpected finding with respect to IL-17A, which has proinflammatory properties. This paradoxical result can be explained by that study’s cross-sectional design and a relatively lower number of subjects. Unlike that study, we detected higher IL-17A level among patients with isolated metabolic syndrome. Thanks to its marked proinflammatory properties, IL-17A may play an important role for the pathogenesis of the metabolic syndrome. This was evidenced by Ohshima et al. who found that IL-17 induced insulin resistance through type 1 receptors of angiotensin 2 in mice [31]. This is because IL-17 also induces the production of IL-6 and TNF-alpha, the cytokines with marked proinflammatory effects that play an important role for the development of insulin resistance [31]. Another study showed that IL-17 play a role for adipocyte differentiation and glucose hemostasis [32]. In that study glucose tolerance and insulin sensitivity was increased among young mice with experimentally created IL-17 deficiency [32]. Hence, IL-17 appears to play a role for glucose metabolism and insulin resistance. Our finding that the patients with metabolic syndrome had a higher IL-17A level also corroborates this notion. Another finding of our study is a significant positive correlation between EAT and IL-17A. As is known, adipocytes may synthesize many cytokines. Additionally, IL-17 has also been shown to play a role for adipocyte differentiation [32]. That is, EAT may exert some of its proinflammatory properties through IL-17. However, this view is only a hypothesis for now because there is no literature information related to the association of EAT and IL-17A. There is a need to conduct experimental and clinical research in this field, including the demonstration of IL-17 expression in EAT. On the other hand, there is strong evidence that IL-17A plays a role in the pathogenesis of atherosclerosis [33,34]. IL-17 expression has been shown in human atheroma plaques [33]. Moreover, it has been reported that some of the IL-17 family are expressed in inflammatory cells infiltrating vascular wall, suggesting that IL-17 family may play a role for the development, progression and complications of atherosclerosis [34]. The most important complication of atherosclerosis is rupture of vulnerable plaques and associated acute coronary syndrome. In this regard, Hashmi et al. reported that serum IL-17A level was increased among patients with acute coronary syndrome [35]. Based on this information, IL-17A may play a role for the conversion of stable coronary plaques into vulnerable plaques with a tendency to rupture. Alteration of macrophage polarization in favour of inflammation also facilitates the conversion of coronary atheroma plaques to vulnerable plaques. That is because macrophages play an important role by synthesizing many proinflammatory cytokines and producing mediators such as matrix metalloproteinases. These data obtained from recent studies suggest that IL-17 plays a role in many stages of atherosclerosis [36,37,38]. We detected an increased serum IL-17A and a positive correlation between serum IL-17A level and EAT, which may be interpreted as an indirect sign of increased coronary artery disease risk among a metabolic syndrome population. Another finding of our study is that an EATT value of 4.7 mm was the cut-off point for prediction of metabolic syndrome. However, the specificity and sensitivity of this cutoff value were relatively low. Bertaso et al. reported that, based on the available data, EATT values above 5 should be considered abnormal [14]. We found a somewhat lower value (4.7 mm). An additional point to consider in this sense is that ethnic origin and racial differences may alter EATT. In conclusion, our cutoff value only applies for the Turkish population. On the other hand, since EAT measurement by echocardiography, which is a simple, inexpensive, and noninvasive method, and an EAT level above this cutoff level would suggest metabolic syndrome among asymptotic individuals, a public health benefit may be obtained by echocardiographic examination of EATT.

Our study has some limitations. A small sample size is it’s main limitation. However, the main reason of this is the exclusion of diabetic and coronary artery disease patients. That is, our study included patients with metabolic syndrome alone. Another important limitation of our study is the measurement of epicardial adipose tissue by echocardiography alone, despite the latter appearing correlated to other imaging methods such as MRI and CT. Moreover, determination of serum Il-17A level by a single measurement and lacking serial measurements and long-term follow-up are the other limitations. On the other hand, we measured Il-17A level from the serum, that is we performed a systemical measurement. Hence, this measurement does not reflect IL-17A’s local and paracrine effects. Additionally; the high IL-17A serum level could reflect as a result of the cumulative effect of other metabolic tissue, such as; visceral adipose tissue and liver. Furthermore, the lack of an assessment of signs of subclinical atherosclerosis such as carotis intima media thickness or coronary calcium score among patients with metabolic syndrome may be considered another limitation. Finally, the lack of measure of other additional proinflammatory cytokines and adipokines could be considered as an important limitation of our study.

In conclusion, epicardial adipose tissue and IL-17A may be associated with the development of insulin resistance, coronary atherosclerosis, and type 2 DM due to their proinflammatory properties among patients with metabolic syndrome. More comprehensive studies are needed to determine the relationship between epicardial adipose tissue and IL-17A more precisely among patients with metabolic syndrome.

## Figures and Tables

**Table 1:** Comparison of the demographic and clinical properties and laboratory results between the metabolic syndrome and control groups.

Parameter	Metabolic Syndrome Group	Control Group	*p* Value
	Mean ± Standard Deviation	Mean ± Standard Deviation	
Age, years	39.0 ± 11.5	39.6 ± 9.4	0.747
Sex (Male/Female)	23/57	16/64	0.197
BMI, kg/m^2^	34.8 ± 6.7	25.9 ± 4.9	<0.001
Smoking, *n*	30	24	0.316
Hypertension, *n*	50	4	<0.001
Hyperlipidemia, *n*	11	4	0.058
Family history, *n*	50	25	<0.001
Systolic blood presure, mmHg	133.8 ± 22.4	111.3 ± 13.4	<0.001
Diastolic blood pressure, mmHg	83.8 ± 12.4	71.3 ± 9.6	<0.001
Glucose, mg/dL	96.4 ± 11.7	84.9 ±10.3	<0.001
Urea, mg/dL	26.8 ± 10.4	25.1 ± 6.8	0.246
Creatinine, mg/dL	0.7 ± 0.2	0.7 ± 0.4	0.536
T. Chol, mg/dL	189.7 ± 38.6	183.1 ± 39.5	0.077
LDL, mg/dL	110.4 ± 29.8	106.3 ± 33.9	0.102
HDL, mg/dL	44.7 ± 11.2	51.9 ± 11.9	<0.001
TG, mg/dL	183.3 ± 91.0	108.7 ± 67.6	<0.001
GGT, U/L	28.9 ± 19.3	20.5 ± 17.0	<0.001
AST, U/L	27.5 ± 17.1	23.8. ± 18.7	0.007
ALT, U/L	33.0 ± 28.1	23.8 ± 33.5	<0.001
Insulin, mIU/ mL	22.0 ± 9.6	18.5 ± 10.3	<0.001
HOMA-IR	5.2 ± 2.3	1.4 ± 0.6	<0.001
IL-17A, pg /mL	2.9 ± 3.7	0.1 ± 0.2	<0.001
hs-CRP, mg/L	3.8 ± 0.6	0.8 ± 0.8	<0.001
EATT, mm	6.2 ± 2.2	4.0 ± 0.8	<0.001
Hb, g/dL	13.1 ± 1.8	12.7 ± 1.6	0.169
Thrombocyte count (x10^9^)	286.9 ± 110.9	257.1 ± 64.1	0.069

BMI, body mass index, T.Chol, Total cholesterol, LDL: ow density lipoprotein, HDL, high density lipoprotein, TG, triglyceride, GGT, Gamma-glutamyl transferase; AST, aspratate aminotransferase; ALT, alanine aminotransferase; HOMA-IR, homeostatic model assessment-insulin resistance; IL-17A; Interleukin 17A; hsCRP, high sensitive C-reactive protein; EATT, epicardial adipose tissue thickness.

**Table 2:** Correlation analysis between EATT and the other parameters.

Parameter		Age	BMI	Glucose	İnsulin	LDL	TG	HDL	GGT	HOMA-IR	hs-CRP	İL-17A	Urea	SBP	DBP
**EATT**	r	0.180	0.539	0.194	0.556	0.092	0.279	-0.142	0.281	0.567	0.666	0.308	0.181	0.421	0.394
	*p*	0.023	0.000	0.014	0.000	0.248	0.000	0.074	0.000	0.000	0.000	0.000	0.022	0.000	0.000

EATT, epicardial adipose tissue thickness; BMI, body mass index; LDL, low density lipoprotein; TG, triglyceride; HDL, high density lipoprotein GGT, Gamma-glutamyl transferase; HOMA-IR, homeostatic model assessment-insulin resistance; hs-CRP, high sensitive C-reactive protein; IL-17A; Interleukin 17A; SBP, systolic blood pressure; DBP, diastolic blood pressure.

**Table 3:** Multivariable Linear Regression Analysis to determine the Effects of Independent Variables on EATT.

Multivariable Model	B	Standard Error	Beta	t	*p*
Age	0.00	0.00	0.18	2.70	0.008
Diastolic blood pressure	0.00	0.00	0.22	3.06	0.003
HOMA-IR	0.03	0.01	0.40	5.65	0.000
urea	0.00	0.00	0.17	2.58	0.011
